# ASD-YOLO: a lightweight network for coffee fruit ripening detection in complex scenarios

**DOI:** 10.3389/fpls.2025.1484784

**Published:** 2025-02-10

**Authors:** Baofeng Ye, Renzheng Xue, Haiqiang Xu

**Affiliations:** ^1^ School of Computer and Control Engineering, Qiqihar University, Qiqihar, China; ^2^ Heilongjiang Key Laboratory of Big Data Network Security Detection and Analysis, Qiqihaer University, Qiqihar, China

**Keywords:** object detection, YOLOv7, attention mechanism, coffee fruit, smart agriculture

## Abstract

Coffee is one of the most popular and widely used drinks worldwide. At present, how to judge the maturity of coffee fruit mainly depends on the visual inspection of human eyes, which is both time-consuming and labor-intensive. Moreover, the occlusion between leaves and fruits is also one of the challenges. In order to improve the detection efficiency of coffee fruit maturity, this paper proposes an improved detection method based on YOLOV7 to efficiently identify the maturity of coffee fruits, called ASD-YOLO. Firstly, a new dot product attention mechanism (L-Norm Attention) is designed to embed attention into the head structure, which enhances the ability of the model to extract coffee fruit features. In addition, we introduce SPD-Conv into backbone and head to enhance the detection of occluded small objects and low-resolution images. Finally, we replaced upsampling in our model with DySample, which requires less computational resources and is able to achieve image resolution improvements without additional burden. We tested our approach on the coffee dataset provided by Roboflow. The results show that ASD-YOLO has a good detection ability for coffee fruits with dense distribution and mutual occlusion under complex background, with a recall rate of 78.4%, a precision rate of 69.8%, and a mAP rate of 80.1%. Compared with the recall rate, accuracy rate and mAP of YOLOv7 model, these results are increased by 2.0%, 1.1% and 2.1%, respectively. The enhanced model can identify coffee fruits at all stages more efficiently and accurately, and provide technical reference for intelligent coffee fruit harvesting.

## 1 Introduction

As one of the three major beverages in the world, coffee is popular with cocoa and tea as the main beverage in the world. China has become the world’s seventh largest coffee consumer. When ripe coffee fruits are not picked in time, they can spoil, leading to economic losses (Vegro and de Almeida, 2020). At present, coffee fruit picking in China mainly relies on manual labor. Due to the four stages of coffee fruit: over-ripe, semi-ripe, ripe and over-ripe (Dalvi et al., 2013), it often leads to low picking efficiency and high cost, which restricts the development of coffee industry. Automated detection of coffee fruit ripened not only prevents wrong picking and reduces costs, however also improves resource utilization. Therefore, automated maturity detection has become an inevitable trend in the development of coffee industrialization.

In recent years, with the rapid development of artificial intelligence, computer vision technology has been widely applied to agriculture, and a large number of scholars have conducted a lot of research in fruit recognition and detection tasks. Fruit identification methods can be broadly divided into two main categories. One category is the traditional image processing methods, which are mainly based on fruit features such as color, texture or shape. It is difficult for such methods to achieve the desired accuracy because they are complicated by factors such as weather effects and occlusion of fruit leaves. The second type of methods are deep learning based methods, It includes single-stage algorithms represented by YOLO (You Only Look Once) (Redmon et al., 2016) series and two-stage algorithms represented by RCNN (region-based convolutional neural networks) (Ren et al., 2016) (Cai and Vasconcelos, 2018) series. For example, based on YOLOX, a SDNet for strawberry maturity detection was proposed by An et al. (2022). He replaced the original CSP block in the backbone network with the self-designed feature extraction module C3HB block, then embedded NAM in the neck, and used the latest SIOU target loss function to improve the accuracy, and finally realized the monitoring of five growth states of strawberry fruit. For example, based on YOLOv5s, a YOLO-CIT for citrus maturation detection at different stages was proposed by Wang et al. (2024), and he proposed R-LBP, which uses Ghostconv instead of traditional convolution. The model can identify citrus at different ripening stages accurately and quickly in real environment. For example, based on YOLOv7-tiny, a YOLO-LM for camellia fruit maturity detection was proposed by Zhu et al. (2024). He introduced three CCA modules into the backbone network and, in addition, introduced GSConv to replace the standard convolution of the Neck network. This model can improve the accuracy of detecting the maturity of camellia fruit in orchard environment. In practice, the one-stage algorithm is more appropriate than the two-stage algorithm because the one-stage algorithm effectively solves the slow speed disadvantage of the two-stage algorithm while maintaining the accuracy.

Many lightweight CNN models have been proposed. Lightweight models reduce the number of parameters however result in a decrease in accuracy. To compensate for the accuracy loss of the lightweight model, the attention mechanism can be used to assign different weights to each part of the input feature layer, extract the basic features, and improve the classification performance. Through the continuous development of YOLO, domestic and foreign scholars have realized the identification and detection of small and densely distributed coffee fruits (Bazame et al., 2021, Bazame et al., 2022). For example, based on YOLOv3-tiny, a method for coffee fruit classification and detection was proposed by Bazame et al. (2021). When the input drawing resolution was 800×800, the model performance reached the highest accuracy in about 3300 iterations. Because of its high calculation time, it is difficult to apply to practical applications. Unal et al. (2022) Four different convolutional neural network-based models (SqueezeNet, Inception V3, VGG16 and VGG19) were used to classify three different coffee beans by transfer learning method, and the experimental results showed that SqueezeNet was the most successful model. Ji et al. (2024) Using hyperspectral imaging to detect coffee beans, two classifier methods are proposed, one combining CEM and support vector machine (SVM) for classification, and the other combining convolutional neural network (CNN) and deep learning for classification. It provides advantages for future practical application and commercialization process. For example, based on YOLOv8n, a model enhanced by WIoUv3, ECA, and C3Ghost for green coffee bean detection was proposed by Ji et al. (2024), achieving higher accuracy. In summary, these methods have made great progress in the object detection of coffee-related tasks through the use of deep learning, however there is still room for improvement in dealing with data diversity, improving model generalization ability, and optimizing resource efficiency. Despite the rapid development of computer vision techniques, only a few studies have been conducted to identify coffee fruits and classify their ripening stages.

In order to improve the detection efficiency of coffee fruit ripening, an improved detection method based on YOLOV7 was proposed to enhance the detection accuracy of coffee fruit ripening.

The main contributions of this paper are as follows:

1. We propose a lightweight one-stage CNN model based on YOLOv7, called Attention SPD-Conv with Dysample YOLO network (ASD-YOLO), for coffee fruit maturity recognition. The SPD-Conv module is used to enhance the detection ability of small fruits and fruits occluded by leaves. We replaced upsampling with Dysample, effectively reducing the number of FLOPs.
2. Due to the high computational cost of dot-product attention, it is difficult to apply to practical applications. We address this problem by proposing a novel Attention mechanism called L-Norm Attention. The new attention is mathematically equivalent to dot-product attention, and the complexity is reduced from $O(n^{2})$ to O(n) . We successfully improve the detection accuracy and computational efficiency of the model.

## 2 Related work

### 2.1 Machine learning methods

The application of machine learning methods to fruit recognition usually consists of three steps: image preprocessing, feature extraction, and fruit recognition. Firstly, the image was converted from RGB (Red, Green, Blue) color to HSV (Hue Saturation Value) and LUV (CIELUV), and grayscale adjustment and noise reduction were carried out. During preprocessing, the background is usually removed. After that, statistical learning methods are used to extract fruit features, and finally machine learning models are used to identify coffee fruits, such as k-means, Support Vector Machine (SVM) and Random Forest (RF) (García et al., 2019; Velásquez et al., 2021), which are used as classifiers to detect coffee fruits. Although the computational cost of this type of algorithms is relatively lower compared with deep learning, the feature extraction is complex and it needs expertise.

### 2.2 Deep learning methods

Because deep learning effectively solves the problem of slow speed while maintaining accuracy, deep learning is widely used in agriculture. Researchers have conducted many studies on coffee fruit recognition and ripeness using deep learning. For example, based on CNNs, a model for coffee classification was proposed by Fuentes et al. (2020), achieving an accuracy of 97.6% with 41 out of 42 tests classified correctly. For example, based on YOLOv4, a high-efficiency, low-cost, and high-precision method for coffee fruit maturity detection was proposed by Bazame (2021). For example, using YOLOv3, a method for identifying coffee and cherries was proposed by Valles-Coral et al. (2023), reducing the running time by three times while maintaining accuracy. For example, combining YOLOv3 with MobileNetv2, a coffee leaf detection method was proposed by Javierto et al. (2021), utilizing a lightweight depth convolution in the intermediate expansion layer to filter nonlinear source features. These studies show that compared with traditional machine learning models, models using the YOLO series can achieve higher accuracy and faster speed in coffee fruit recognition and ripened detection tasks.

### 2.3 The attention mechanism

In recent years, attention mechanism has been widely used in computer vision. Although the introduction of attention mechanisms can help deep learning methods focus on important information, some attention methods are computationally expensive and difficult to implement in practice. For example, Non-local (Wang et al., 2018) can solve the receptive field problem to a large extent, however it is severely limited in computational complexity. In order to reduce the amount of computation, the simplest method is to reduce the number of channels and reduce the resolution, while these methods will cause a decrease in accuracy. CCNet (Huang et al., 2019) is different from Non-Local, which computes the attention of the whole graph at once. The complexity is effectively reduced by Criss-cross attention. Efficient Attention (Shen et al., 2021) achieves linear complexity by clever use of *Softmax*. ANNNet (Zhu et al., 2019) provides sample in terms of calculating key and value, which reduces the size of key and value, thus reducing the amount of calculation when performing *Softmax* operations. DANet (Fu et al., 2019) introduces spatial attention and channel attention at the same time, which effectively expands the receptive field, however the high amount of calculation generated is not friendly to GPU devices.

## 3 Materials and methods

### 3.1 Coffee fruit ripeness object detection dataset

**Figure 1:**
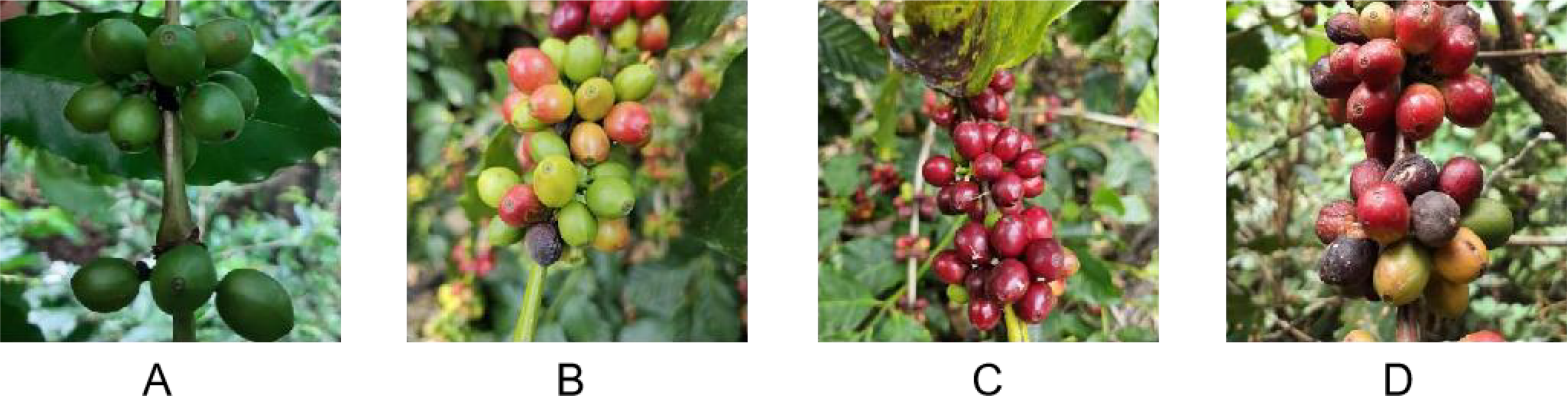
Representative images of over-ripe, ripe, semi-ripe and unripe coffee fruits. **(A)** unripe; **(B)** semi-ripe; **(C)** ripe; **(D)** over-ripe.

Our dataset comes from contributions from roboflow. Coffee fruit maturity was divided into four categories, including over-ripe, ripe, semi-ripe and unripe. The background of the coffee fruit is affected by the shadow and the influence of the occlusion of the leaves, which has a complex light environment. This mimics real application scenarios and may enhance the robustness of the trained model. There were significant differences in the characteristics of fruit maturity among the four coffee varieties. **Figure 1** shows representative images of the four categories. We divide the dataset into, train valid and test datasets, corresponding to 4359, 425 and 198 images per group, respectively. These images have uneven distribution of fruit natural scenes such as fruit occlusion, overlapping occlusion, branch occlusion, visual appearance similar to the background image, and dense targets.

### 3.2 ASD-YOLO

YOLO stands for “You Only Look Once” and is a fast and accurate object detection algorithm. YOLO v7 uses a large number of ELAN as the base module. The multiple stacking corresponds to a denser residual structure. The characteristics of the residual network are easy to optimize, and it can improve the accuracy by increasing a certain depth. YOLOv7 has different variations, such as YOLOv7-Tiny,YOLOv7 and YOLOv7-w6.

In agricultural applications, YOLO series models have significant advantages in the field of object detection, especially in crop monitoring, pest detection, crop classification, fruit maturity judgment and other tasks. YOLOv7 reduces the complexity of the model, making the inference relatively fast. This is especially important for real-time inspection in agricultural applications. YOLOv10 and YOLOv11 have advantages in detection accuracy on high-resolution images. However, in today’s agricultural applications, most of them are faced with the challenges of complex scenes and low image resolution, YOLOv7 still performs well in terms of speed and accuracy, and its low computing overhead and high reasoning speed are very suitable for YOLOv7. YOLOv9, YOLOv10 and YOLOv11 may be suitable for some complex missions. For example, YOLOv7-BiGS for real-time citrus identification was proposed by Deng et al. (2024), meeting the real-time requirement while effectively ensuring accuracy. For example, YOLO-SwinTF for tomato detection was proposed by Liu et al. (2024), achieving excellent performance in detection accuracy while maintaining a comparable detection speed. For example, YOLOv7-E for accurate inflorescence detection and positioning at different distances was proposed by Zhang et al. (2024), providing effective technical support for future fruit thinning machinery in differentiation and precise flower thinning operations.

In general, YOLOv7 network is divided into four parts: Input, backbone network, neck network and head network. The size of the input image in the YOLOv7 model is 640×640. The YOLOv7 model uses 3×3 or 1×1 convolution cores. These convolution kernel sizes are chosen based on empirical evidence and computational considerations. These 3×3 convolution cores capture spatial information for local regions in the input image, while 1×1 convolution cores perform channel-level operations to adjust the depth of the feature mapping. Firstly, the images are preprocessed by operations such as data augmentation in the input part, and then fed into the backbone network. The backbone network framework is constructed by convolution, E-ELAN module, MPConv module and SPPCSPC module. The backbone network is responsible for feature extraction of the input image, and then the extracted feature map is enhanced by the Neck module. The Neck module aggregates low-level spatial features and high-level semantic features through FPN+PAN, which aims to combine feature information at different scales. It significantly improves the accuracy of recognizing objects at different scales. Finally, the head generates the object bounding box with coordinates, category, and confidence.

**Figure 2:**
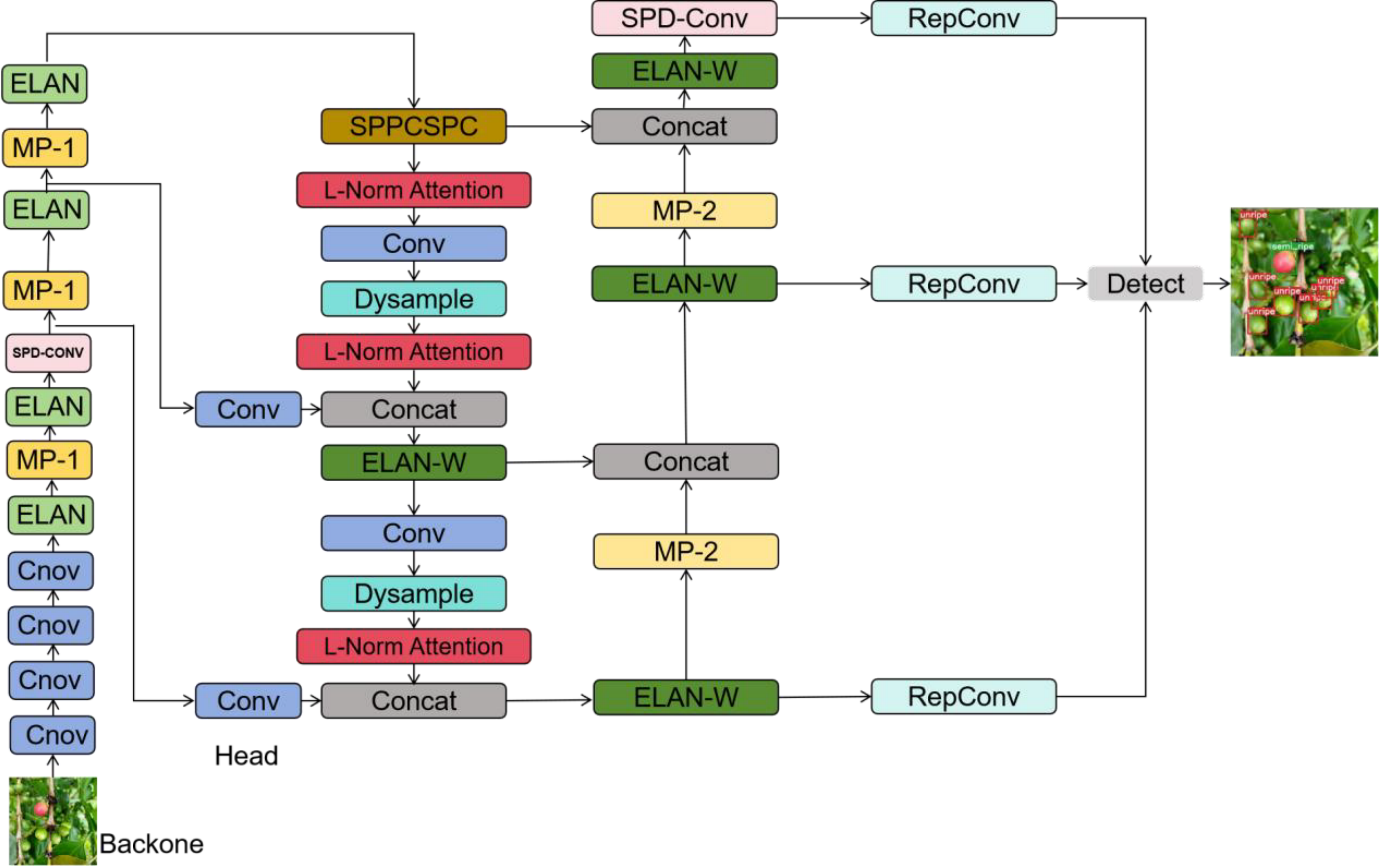
ASD-YOLO network structure.

The network structure of ASD-YOLO is mainly composed of two parts: Backbone and Head. The extraction of image features is mainly implemented in backbone. SPD-Conv (Sunkara and Luo, 2022) is introduced into backbone and head to enhance the pair of small fruits and fruits occluded by leaves. In order to avoid generating a large number of parameters, we only introduce one layer in backbone and head. After that, we add our designed L-Norm Attention to the head to enhance the feature extraction ability of the neural network model through the attention mechanism. Finally, we replace the upsampling in head with Dysample (Liu et al., 2023), which reduces the computational load of the model while improving the image resolution. The network structure of ASD-YOLO is shown in **Figure 2**.

The Backbone network is the feature extraction part of the ASD-YOLO model, which extracts high-level features from raw images. The Backbone network here consists of a series of convolution layers, pooling layers, and a SPD-Conv layer. The layers are stacked in order. The conv module consists of convolution, batch normalization, and SiLU activation functions to extract features. The Backbone network starts with a convolution layer with 3×3 kernels and step size 1, which is used to process the input image. In the following layers, the Backbone network gradually increases the feature depth through convolution layers. These convolution layers include layers with different numbers of filters and different sizes of convolution kernels to gradually extract more complex features. After some convolution layers, the backbone network includes a Maxpooling layer. To address the fine-grained information lost using the conv step and/or pooling layers, we use the SPD-Conv module to handle it, especially on small objects and low-resolution images.

The Head network is the output generation part of the ASD-YOLO model, which converts the feature maps extracted from the backbone network into outputs for object detection. The Head network consists of a series of convolution layers, Dysample layers, concatenation layers, SPD-Conv layers and L-Norm attention layers, as well as an object detection layer. The Head network consists of multiple convolution layers with different number of filters and different kernel sizes, and these layers are used to process the feature maps from the neck network. To address the loss of fine-grained information introduced by using conv step and/or pooling layers, we add a layer of SPD-Conv to head. The Head network zooms in on the feature maps on the upsampling layers to merge them with the feature maps at different scales in the Neck network. Feature maps from different layers of the neck and head networks are merged by concatenation to combine multi-scale information. L-Norm Attention is an attention module designed by ourselves. We also replaced upsampling with DySample, which does not require a custom CUDA package and has far fewer parameters, FLOPs, GPU memory, and latency. Detect is the last layer of the head network and is used to generate the output of object detection. It accepts feature maps from different scales and uses anchors for object detection, generating detection boxes along with the corresponding category confidence and location information for each box.

### 3.3 The L-Norm Attention module

The texture features of coffee fruit have the characteristics of low resolution, small pixel area and small object. We enhance the extraction of coffee fruit features by adding L-Norm Attention. From recent studies, the performance of dot product attention mechanism in image processing tasks is getting better and better. However, we observe the drawback of the dot product attention: the high computational complexity is difficult to apply in practice. We propose an efficient attention mechanism that is equivalent to the dot product attention, however achieves the desired level of complexity o(n) . Where *Q* (Query) refers to the scope of the query, autonomous suggestion, that is, the feature vector of subjective consciousness, *K* (Key) refers to the item being compared, non-autonomous suggestion, that is, the prominent feature information vector of the object, and *V* (Value) is the feature vector representing the object itself, which usually appears in pairs with Key. The form of dot product attention is shown in Equation 1:


(1) $$\text{Attention}(Q,K,V)\;=\text{softmax}(QK^{T})V$$

We observe that the key factor limiting the performance of dot product attention is *Softmax*. This step yields an n×n matrix of, and it is this step that makes the dot product attention complexity $o(n^{2})$ . That is, removing *Softmax* reduces the complexity to linear. We can come up with a general definition of Attention: that is, replace $\;QK^{T}\;$ with the general function sim ( $q_{i},k_{j}$ ) of $q_{i},k_{j}$ . Then it is sim ( $q_{i},k_{j}$ ) = $QK^{T}$ . We define the equivalent formula for the dot product attention as Equation 2:


(2) $$\text{Attention}{\left(Q,K,V\right)}_{i}=\frac{\sum_{j=1}^{n}sim(q_{i},k_{j})v_{j}}{\sum_{j=1}^{n}sim(q_{i},k_{j})}$$

Removing *Softmax*, it can be expressed as Equation 3:


(3) $$sim(q_{i},k_{j})\;=\;q_{i}^{T}k_{j}$$

The question is how to satisfy $\text{sim}({\text{q}}_{\text{i}},{\text{k}}_{\text{j}})\geq 0$ . Efficient Attention uses a flexible way to perform *Softmax* on *Q* and *K* separately, instead of performing *Softmax* after the operation. Our idea is to cancel *Softmax* and add 1 after L2 normalization of *Query* and *Key*. It can be expressed as Equation 4:


(4) $$sim(q_{i},k_{j})\;=\;1\;+\;L2Norm{\left(q_{i}\right)}^{T}L2Norm(K_{j})$$

**Figure 3:**
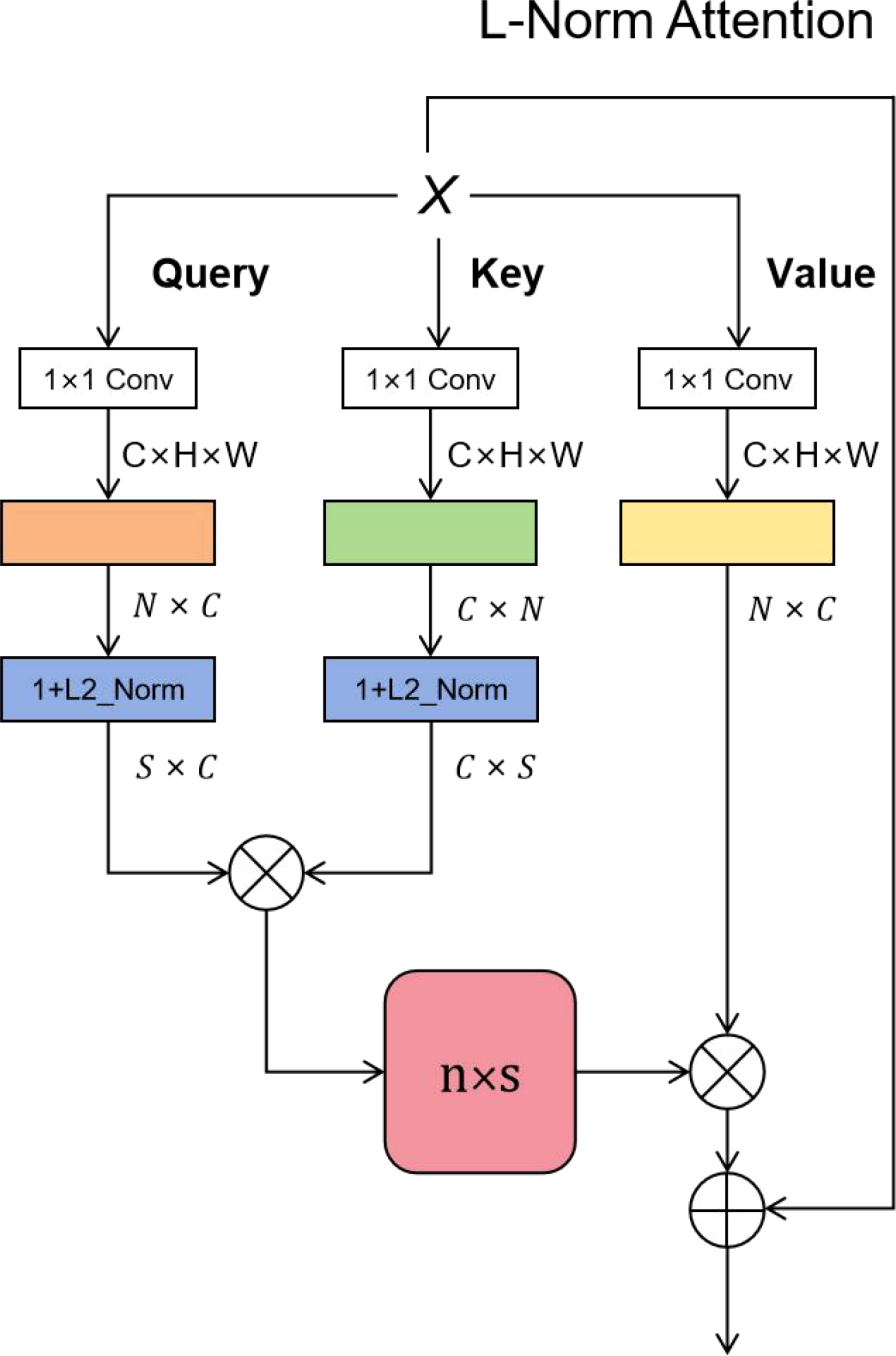
⊗ denotes matrix multiplication and ⊕ denotes addition, where s≤n . Our designed attention mechanism is mathematically equivalent to and approximately equivalent to the dot product attention.

As shown in **Figure 3**, for the input feature map, it is changed into a matrix, and after L-Norm Attention, it is changed to h×w×d again. Finally, the output features are added to the input features to form a residual structure.

### 3.4 The SPD-Conv module

**Figure 4:**
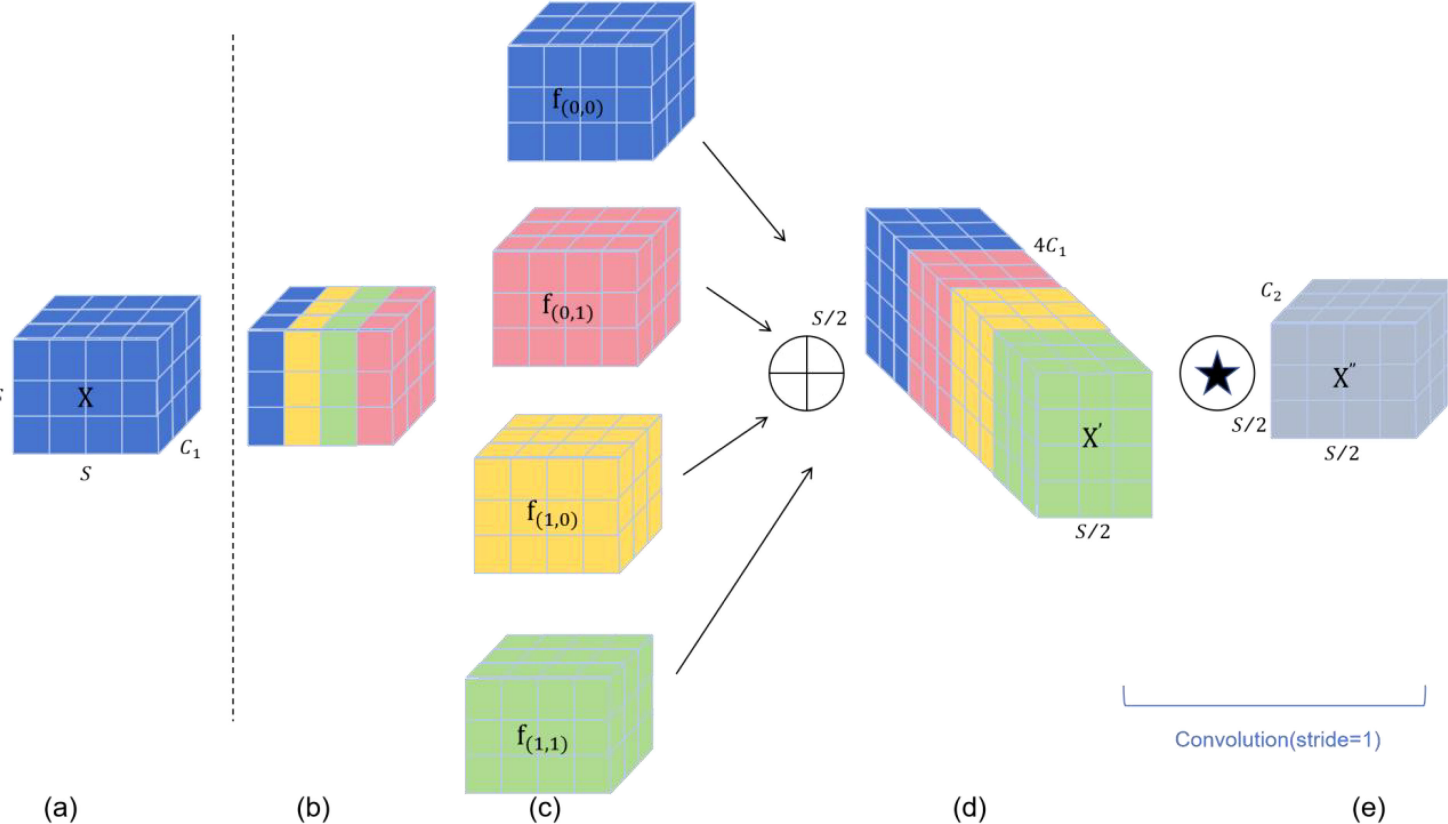
The SPD-Conv module. **(A)** shows the standard feature map, **(B)** is the space-to-depth operation, **(C)** shows the depth increase of the resulting feature map, **(D)** shows the non-step convolutional layer applied after the SPD operation, and **(E)** shows the output feature map after a convolution with step 1 that preserves spatial resolution however changes the depth dimension.

In object detection, we have observed that convolution neural Networks (CNNs) have achieved excellent results in image processing tasks, however, when the resolution of the image being detected is low or the object being detected is small, their detection accuracy will be affected. The ultimate reason is that some fine-grained information is lost when using conv step and pooling layers. Therefore, we are compensating for this loss of fine-grained information by adding the SPD-Conv module to backbone and head. SPD-Conv consists of a space-to-depth (SPD) layer and a convolution-free step (Conv) layer, as shown in **Figure 4**. Firstly, the spatial dimension of the input image is converted into the depth dimension, which avoids the information loss in the traditional step convolution and pooling operations. Thus, the depth of the feature map can be increased without losing information, and more spatial information can be retained through this layer. After that, by applying a convolution layer, no step size is used. This is because the non-step convolution is able to perform feature extraction without reducing the size of the feature map, which further preserves the fine-grained information of the image. This combination of the combined use of SPD layers and non-step convolution layers enables the CNN to better detect small objects and low-resolution images, improving the performance and robustness of the model in these complex scenes.

### 3.5 The Dysample module

**Figure 5:**
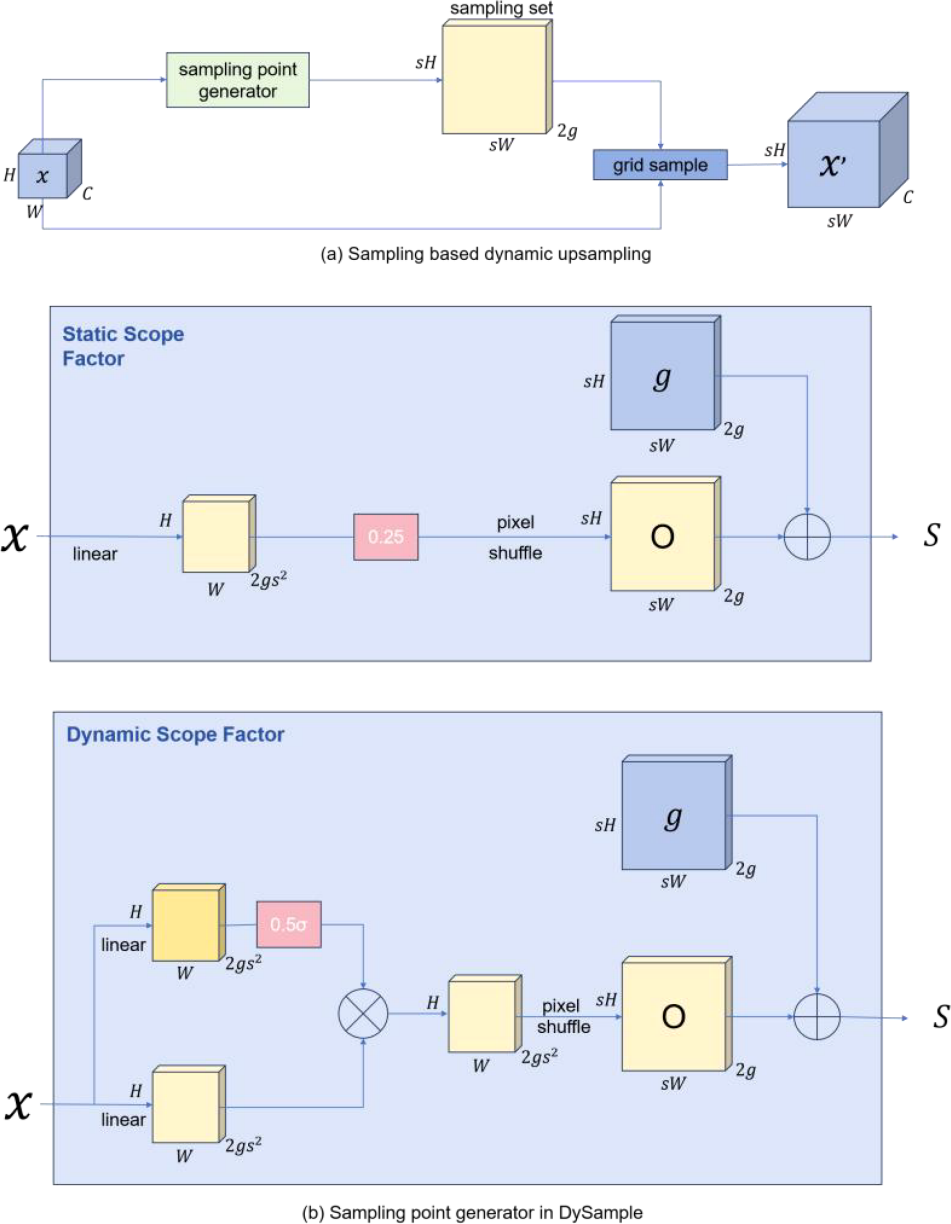
The DySample module **(A)** The sample set is generated by the sample point generator, and the input features are resampled by the grid sampling function. In generator **(B)**, the sampling set is the sum of the generated offsets and the original grid locations.

Feature upsampling is a key component in object detection models to recover feature resolution. The most popular ones are bilinear interpolation and nearest neighbor. They follow fixed rules to interpolate the upsampled values. With the popularity of dynamic networks, some excellent upsamplers have also shown excellent potential. CARAFE (Wang et al., 2019), FADE (Lu et al., 2022) and SAPA, for example, have significant performance gains while also suffer from high computational overhead due to the time consumption of dynamic convolutions and the additional subnetworks used to generate dynamic kernels. In addition, FADE and SAPA require high-resolution feature guidance, which limits their application scenarios to some extent. Dysample bygoes dynamic convolutions and reconstructs the upsampling process from the point sampling perspective, which is more resource efficient and easy to implement. DySample eliminates the need for custom CUDA packages and significantly reduces the number of parameters, FLOPs, GPU memory, and latency. **Figure 5** shows the module diagram of Dysample.

Therefore, we integrate Dysample into our ASD-YOLO network to focus on upsampling low-resolution images to higher resolutions with minimal overhead.

### 3.6 Hardware and software

For the train and test of the research work in this paper, a computer with ubuntu18.04 operating system, 24 GB RAM (NVIDIA GeForce RTX 4090GPU), python 3.8.18 and torch-1.9.0 is used for the experiments. Weights were initialized using a random initialization technique and all models were trained from scratch. **Table 1** shows the hyperparameter settings for the network model.

**Table 1:** Network model hyperparameter settings.

Parameter settings	Illustrate
Epoch	150
Batch size	16
Learning rate	0.01
Weight decay	0.005
Optimizer	SGD
Size	640

### 3.7 Evaluation metrics

The metrics are as follows (Sirisha et al., 2023): precision, recall, and AP. For binary classification problems, samples can be classified into four types: true positive (TP), false positive (FP), true negative (TN), and false negative (FN). Equations 5, 6 for precision (P) and recall (R) are as follows.


(5) $$P\;=\;\frac{TP}{TP\;+\;FP}$$

(6) R = TP/(TP + FN)

Precision is a measure of the relevance of the results, while recall is a measure of how many truly relevant results are returned. Our models should be comprehensively evaluated in terms of detected boundaries and classification performance. The most widely used criterion is the mean Average Precision (mAP) adopted in the following tests as shown in Equation 7. In addition, mAP needs to be evaluated using a threshold IoU. As shown in Equation 8:


(7) $$mAP\;=\;\frac{1}{m}\;\sum \;AP\;(i)$$

(8) $$IoU(m,\;n)\;=\;\frac{area\;(m\cap n)}{area\;(m\cup u)}$$

*m* denotes the truth box and *n* denotes the bounding box. AP50 and mAP are used to evaluate the overall performance of the detection. mAP refers to the average precision value over different IoU thresholds ranging from 0.5 to 0.95.

## 4 Experimental results and discussion

### 4.1 Experimental results

**Figure 6:**
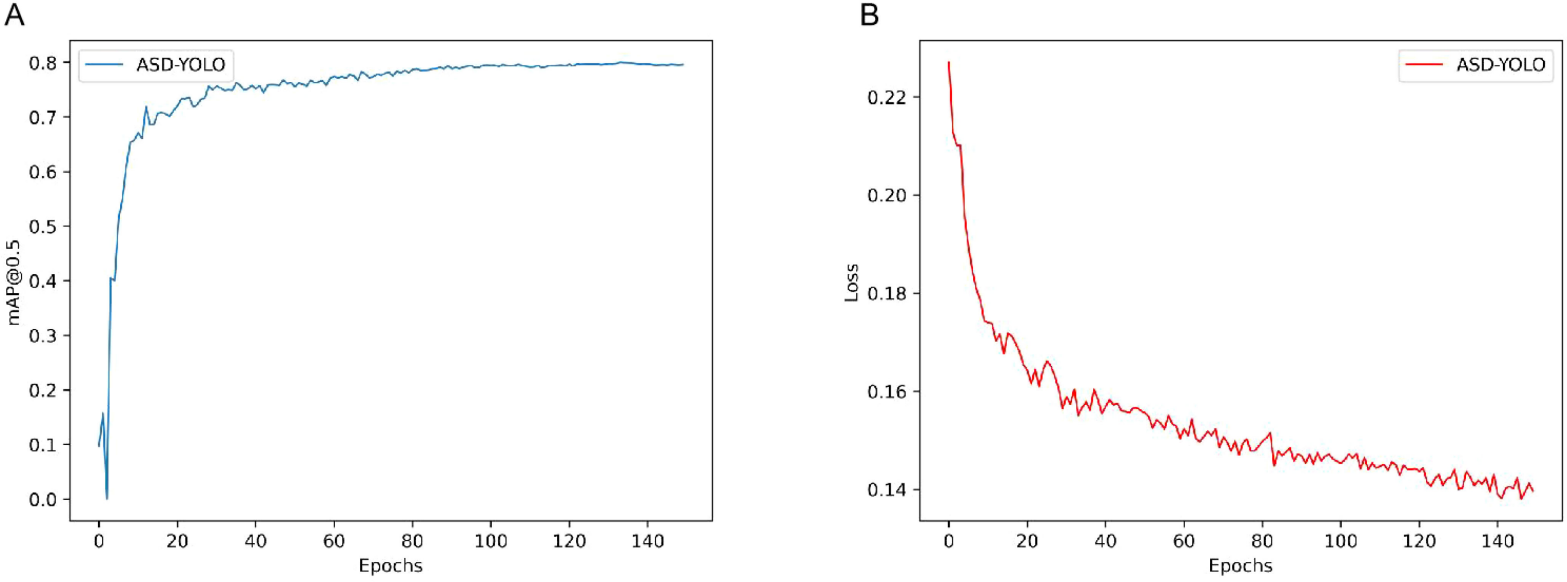
The training outcome of models and Loss map. **(A)** The training outcome of models. **(B)** Loss map for model training and validation.

Deep learning models are often referred to as black boxes because they have complex architectures and a large number of parameters that obscure their internal mechanisms. This lack of transparency poses a significant obstacle to the train and evaluation of these models. In this paper, the training results and the sum loss function of the model are analyzed. As shown in **Figure 6**, the decrease in validation loss correlates with the increase in mPA. **Figure 6B** describes the detailed monitoring of the loss function values throughout the training phase and draws special plots for validation data sets. The trend described in **Figure 6** shows that the consistency of the model converges as the training iteration progresses. As the model continues to learn, its performance steadily improves. As shown in **Figure 6A**. This convergence serves as compelling evidence to support the validity of our model and affirms the validity of our training and evaluation methods.

**Figure 7:**
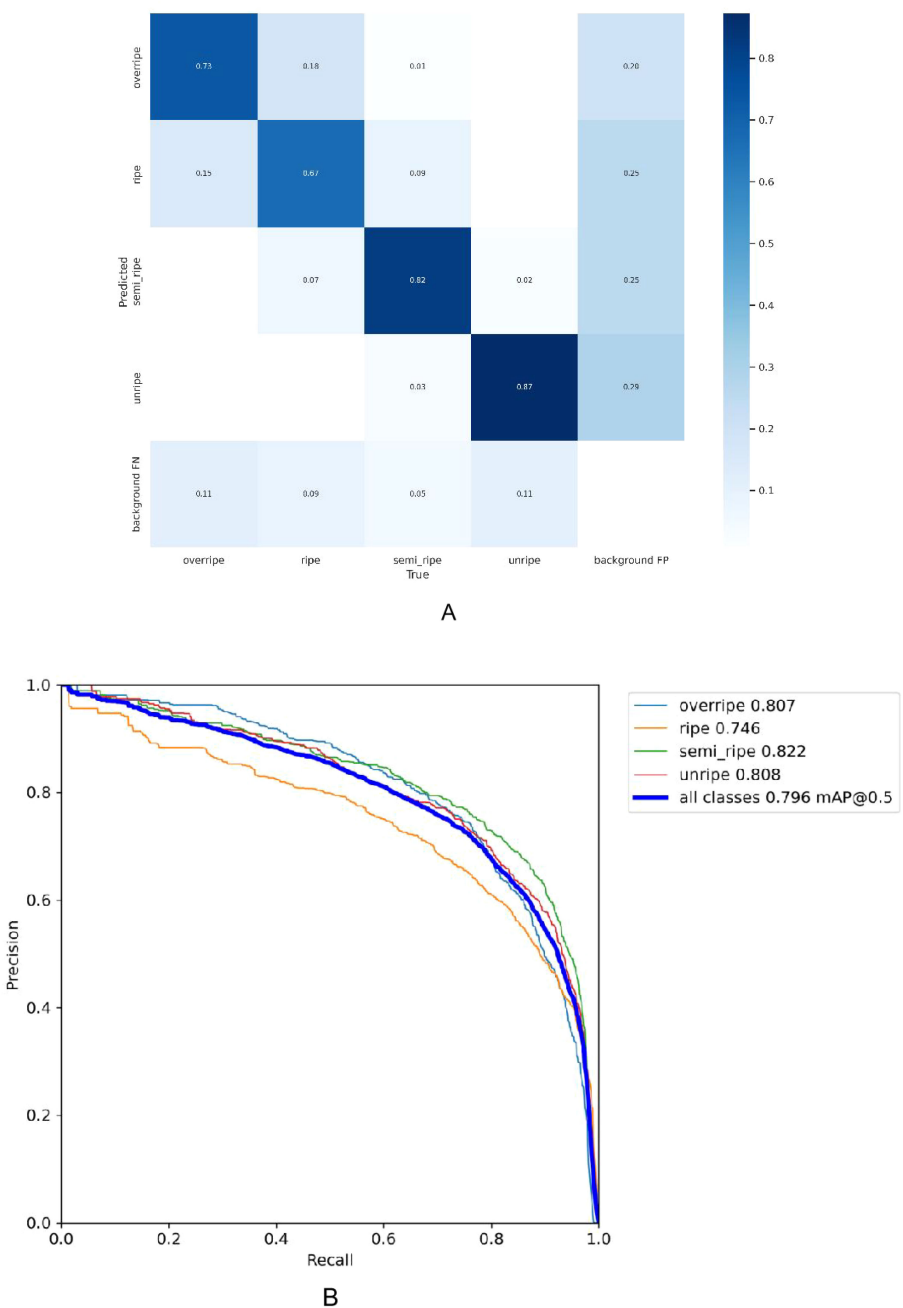
Confusion matrix and precision-recall curve for the model. **(A)** Confusion matrix **(B)** Precision-Recal curve.

Based on the experimental results, **Figure 7A** shows the confusion matrix summarizing the prediction results of the classification. The confusion matrix is a tool for evaluating the performance of a classification model by comparing the model’s predicted class tables with the true data categories. This matrix takes the form of a two-dimensional array, where rows represent the actual categories and columns represent the predicted categories. As observed from the confusion matrix in **Figure 7A**, the values on the main diagonal (0.73, 0.67, 0.82 and 0.87) indicate the proportion of instances correctly predicted by the model for each class. Elements outside the diagonal represent the degree of confusion between different classes by the model. For example, in the unripe class, there is a 2% probability that instances are incorrectly predicted as the semi-ripe class. It can be seen that the prediction of coffee fruit maturity at different stages is excellent. The proportion of false positives is very small, and the occasional false negative cases may be due to the occlusion of fruits and leaves and the influence of complex environmental factors, which may affect the performance of the model.

As shown in **Figure 7B**, the Precision-Recall curve illustrates the P performance of a classification model for each class at different R thresholds. P refers to the ratio of the number of samples predicted as positive and actually positive to the total number of samples predicted as positive by the model, while R refers to the ratio of the number of samples predicted as positive and actually positive to the total number of actual positive samples. Four PR curves are presented in the graph, corresponding to four class tables: over-ripe, ripe, semi-ripe, and unripe. The calculation of AP was performed at a fixed R threshold of 0.5. The curves are located near the upper right corner of the coordinate axes, indicating that the model maintains high P while maintaining a high R rate, demonstrating excellent classification performance on these classes.

#### 4.1.1 Comparison experiments

The results in **Table 2** show that different backbone modules have different influences on the detection results of the original YOLO v7 model. After we introduced the SPD-Conv module into YOLO v7, the accuracy increased by 0.7% to 78.7% when the calculation amount remained almost unchanged and the number of parameters decreased by 3.0%. Specifically, compared with SPPCSPC module and DSConv module, SPD-Conv has the highest improved accuracy. In resource-constrained environments, SPD-Conv improves computational efficiency and inference speed while maintaining reasonable feature representation and detection accuracy.

**Table 2:** Different backbone methods.

Module	mAP@0.5/%	Parameters/M	FLOPS/G	FPS
YOLOv7	78.0	37.2	105.2	114.0
YOLOv7-SPD-Conv	78.7	38.2	102.2	91.7
YOLOv7-DSConv	77.8	37.2	95.7	91.7

**Table 3** shows the comparison results. This article uses DySample to update the upsampling operation of the original model. Compared with the original nearest neighbor interpolation method, the accuracy of DySample is improved by 0.5% under the condition that the calculation amount and parameter number are basically unchanged. In contrast, the lightweight upsampling operator CARAFE improves the accuracy compared to the original upsampling, while the effect is not as good as DySample. Therefore, this paper chooses DySample operator to replace the original upsampling.

**Table 3:** Different upsampling method comparison results.

Upsampling	mAP@0.5/%	Parameters/M	FLOPS/G	FPS
Nearest	78.0	37.2	105.2	114.0
DySample	78.5	37.2	105.2	112.4
CARAFE	78.3	37.5	105.6	106.3

We compared different attention methods based on the YOLO v7 model to verify the validity of our proposed L - Norm attention. We respectively compared SE (Hu et al., 2018), ECA (Wang et al., 2020), CBAM (Woo et al., 2018), CA (Hou et al., 2021), GAM (Liu et al., 2021) and our proposed L - Norm attention. The comparison results are shown in **Table 4**.

**Table 4:** Comparison of different attention mechanisms.

Attention	mAP@0.5/%	Parameters/M	FLOPS/G	FPS
YOLOv7(Base)	78.0	37.2	105.2	114.0
SE	78.2	37.5	105.3	108.6
ECA	78.7	37.2	105.2	100.0
L-Norm Attention	78.9	37.6	105.3	108.7
CBAM	78.6	37.3	105.6	111.3
CA	78.2	37.5	105.5	104.6
GAM	78.3	46.4	114.9	89.1

According to the experimental results in **Table 4**, L-Norm Attention was introduced into YOLO v7, and the accuracy was increased by 0.9%, reaching 78.9%, while the amount of computation and the number of parameters remained almost unchanged. Compared with other kinds of attention, our proposed attention method can significantly improve the accuracy.

We comprehensively evaluated the accuracy of the model using metrics such as Precisio, Recall, and mAP. To demonstrate the excellent performance of our model architecture, we compare our proposed ASD-YOLO method with several SOTA object detection algorithms. **Table 5** shows the experimental results of the improved model and different YOLO models on the coffee fruit maturity dataset. Among them, the YOLO series, along with Faster-Rcnn and Efficientdet, were all tested in one environment. Their epochs are 150, their batch size is 16, and their input size is 640×640. The YOLO Optimizer uses sgd, the Faster-Rcnn uses adam, and the Efficientdet uses adamw; The YOLO series has a weight decay of 0.005 and a Faster-Rcnn of 0.

**Table 5:** Results of ASD-YOLO, YOLOv5, YOLOv7-YOLOv11, Faster-Rcnn and Efficientdet in the test set.

Model	Precision/%	Recall/%	mAP@0.5/%	FLOPS/G	Parameters/M
YOLOv5s	67.0	77.0	78.5	7.2	25.1
YOLOv7	68.7	76.4	78.0	105.2	37.2
YOLOv8n	68.0	78.4	78.7	8.2	30.1
YOLOv9	69.0	76.5	78.5	315.7	706.1
YOLOV10n	70.5	76.4	79.1	8.4	27.1
YOLOV11	70.1	75.4	78.3	6.4	26.0
Faster-Rcnn	47.1	16.3	25.9	137.1	370.2
Efficientdet	–	27.3	26.9	208.2	6.7
ASD-YOLO	69.8	78.4	80.1	59.2	36.3

As shown in **Table 5**, the experimental results of five object detection models, YOLOv5, YOLOv7, YOLOv8, YOLOv9, YOLOv10, YOLOv11, Faster-Rcnn,Efficientdet and ASD-YOLO, are analyzed, and the following observations are made: ASD-YOLO shows excellent performance in precision, recall and mAP@0.5 with values of 0.698, 0.784, 0.801 and 0.732, respectively, which is 1.4% higher than the second-place YOLOv8 on mAP@0.5. Through comparative analysis with other models, ASD-YOLO has excellent accuracy and detection ability. Integrating the L-Norm attention mechanism, SPD-Conv, and Dysample into the YOLOv7 network structure, ASD-YOLO is significantly enhanced. Empirical evidence shows that the modified ASD-YOLO is more resistant to interference and can more reliably detect specific characteristics of coffee fruits with different ripeness. Therefore, it is an effective improvement strategy to integrate the improved L-Norm attention mechanism, SPD-Conv and Dysample into the YOLOv7 network structure.

**Figure 8:**
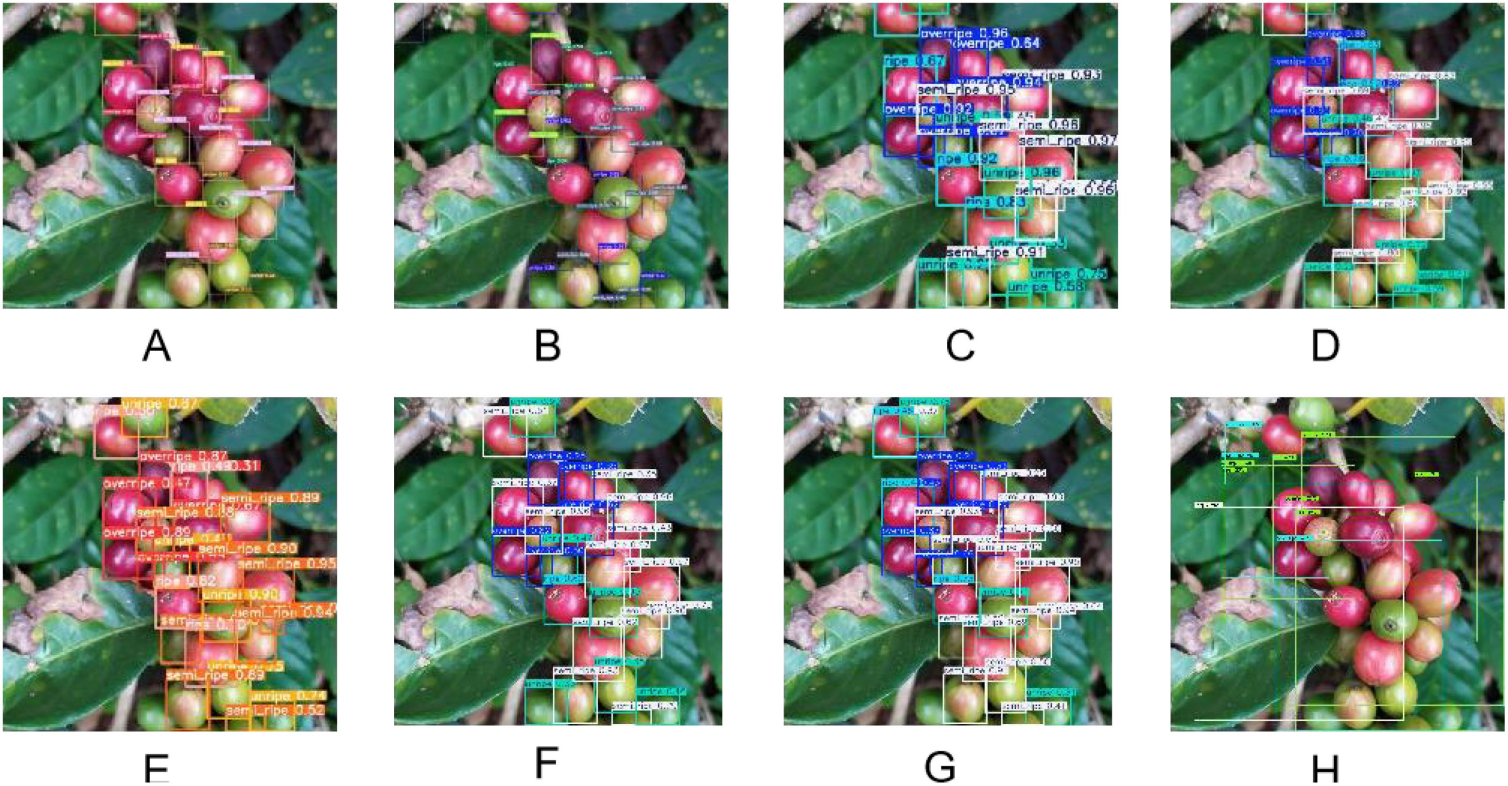
Comparison of coffee fruit detection at different ripeness with occlusion and overlap. **(A)** Display the detection results of ASD-YOLOv; **(B)** Display the detection results of YOLOv7; **(C)** Show the detection results of YOLOv5. **(D)** Shows the detection results of YOLOv8; **(E)** Shows the detection results of YOLOv9; **(F)** is the detection result of YOLOv10, **(G)** is the detection result of YOLOv11 and **(H)** is the detection result of Efficientdet.

The test results are shown in **Figure 8**. ASD-YOLOv7 (**Figure 8A**), YOLOv7 (**Figure 8B**), YOLOv5 (**Figure 8C**), YOLOv8 (**Figure 8D**), YOLOv9 (**Figure 8E**), YOLOv10 (**Figure 8F**), YOLOv11 (**Figure 8G**), effentdet (**Figure 8H**). In the same picture, there are coffee fruits of different maturity with unclear features and blocked features at the same time, as shown in **Figure 8**, over-ripe, ripe, semi-ripe and unripe coffee fruits have small differences, which are difficult to identify easily, and more obvious features are needed to be recognized. This leads to misjudgment of the maturity detection results of YOLOv7. However, the confidence of the improved ASD-YOLO detection results is impressive and can extract features with high confidence, however also identify coffee fruits of similar maturity that are occluded due to fruit overlap. ASD-YOLO can accurately identify coffee fruits with different maturity levels while overcoming the problem of occlusion. However, in practice, unpredictable lighting conditions are difficult to standardize, and these conditions affect the detection of maturity. Therefore, the characteristics of complex scenes are in line with actual application scenarios, and the generalization ability of ASD-YOLO method is well highlighted.

#### 4.1.2 Ablation experiments

Preliminary experiments on the coffee fruit dataset use YOLOv7 as a baseline model. The results show that YOLOv7 performs well in detecting clear, medium, and large objects. However, there is still room for improvement in detecting partial occlusions and objects with indistinct features. Therefore, the attention mechanism is introduced into YOLOv7 to enhance the feature extraction ability of the model.

This paper adopts an approach combining ablation experiments and comparative experiments to validate the effectiveness of the proposed algorithm. Ablation experiments, as depicted in **Table 6**, are conducted to dissect and verify the efficacy of the improvements made. Through the ablation experiment, components were added sequentially and the improved network performance after adding components was compared to verify the necessity of the corresponding improvements. First, SPD-Conv is embedded in YOLOv7-based Backbone and Neck for performance comparison. Second, the Dysample is replaced with the original upsample for performance comparison. Finally, L-Norm attention mechanism is added for performance comparison. The comparison results with the original algorithm YOLOv7 are shown in **Table 6**.

**Table 6:** Results of ablation experiments on the coffee fruit dataset.

Model	Precision(%)	mAP@0.5	mAP@0.5:0.95	FLOPS/G	Parameters/M
YOLOv7	68.7	78.0	73.4	105.2	37.2
YOLOv7+SPD-Conv	68.9	78.7	71.2	102.2	38.2
YOLOv7+SPD-Conv +Dysample	69.3	79.2	73.3	61.7	39.3
YOLOv7+SPD-Conv +Dysample +L-Norm Attention	69.8	80.1	73.2	59.2	36.3

## 5 Conclusions

In this paper, a method for identifying the maturity of coffee fruits is proposed, which can automatically detect coffee fruits with different ripeness and improve the accuracy of coffee fruit detection. We use YOLOv7 as the base network, integrate the improved attention mechanism into head, and refer to SPD-Conv in Backbone and Head, after which all the upsampling in the model is replaced with Dysample. ASD-YOLOv7 successfully completed the maturity detection task and outperformed YOLOv7 in coffee fruit maturity detection performance. The combination of SPD-Conv, L-Norm Attention, and Dysample with YOLOv7 achieves the best performance as demonstrated by ablation experiments.

The results show that the ASD-YOLOv7 model performs well in the coffee fruit maturity detection task, which provides technical support for smart agriculture. In addition, the maturity detection technology can provide a more effective method for the automated picking of coffee fruits.

## Data Availability

The original contributions presented in the study are included in the article/supplementary material. Further inquiries can be directed to the corresponding author.

## References

[B1] An Q. Wang K. Li Z. Song C. Tang X. Song J. (2022). Real-time monitoring method of strawberry fruit growth state based on YOLO improved model. IEEE Access 10, 124363–124372. doi: 10.1109/ACCESS.2022.3220234

[B2] Bazame H. C. (2021). Quantification and classification of coffee fruits with computer vision (Universidade de São Paulo).

[B3] Bazame H. C. Molin J. P. Althoff D. Martello M. (2021). Detection, classification, and mapping of coffee fruits during harvest with computer vision. Comput. Electron. Agric 183, 106066. doi: 10.1016/j.compag.2021.106066

[B4] Bazame H. C. Molin J. P. Althoff D. Martello M. (2022). Detection of coffee fruits on tree branches using computer vision. Sci. Agric. 80, e20220064. doi: 10.1590/1678-992x-2022-0064

[B5] Cai Z. Vasconcelos N. (2018). Cascade r-cnn: Delving into high quality object detection. In Proceedings of the IEEE conference on computer vision and pattern recognition. 6154–6162.

[B6] Dalvi L. P. Sakiyama N. S. Silva F. D. Cecon P. (2013). Quality of coffee cherry and sugarcane-green stage by electrical conductivity.

[B7] Deng F. Chen J. Fu L. Zhong J. Qiaoi W. Luo J. (2024). Real-time citrus variety detection in orchards based on complex scenarios of improved YOLOv7. Front. Plant Sci. 15, 1381694. doi: 10.3389/fpls.2024.1381694 39011299 PMC11246913

[B8] Fu J. Liu J. Tian H. Li Y. Bao Y. Fang Z.. (2019). “Dual attention network for scene segmentation,” in Proceedings of the IEEE/CVF conference on computer vision and pattern recognition. 3146–3154.

[B9] Fuentes M. S. Zelaya N. A. L. Avila J. L. O. (2020). “Coffee fruit recognition using artificial vision and neural networks,” in 2020 5th International Conference on Control and Robotics Engineering (ICCRE): IEEE. 224–228.

[B10] García M. Candelo-Becerra J. E. Hoyos F. E. (2019). Quality and defect inspection of green coffee beans using a computer vision system. Appl. Sci. 9, 4195. doi: 10.3390/app9194195

[B11] Hou Q. Zhou D. Feng J. (2021). “Coordinate attention for efficient mobile network design,” in Proceedings of the IEEE/CVF conference on computer vision and pattern recognition. 13713–13722.

[B12] Hu J. Shen L. Sun G. (2018). “Squeeze-and-excitation networks,” in Proceedings of the IEEE conference on computer vision and pattern recognition. 7132–7141.

[B13] Huang Z. Wang X. Huang L. Huang C. Wei Y. Liu W. (2019). “Ccnet: Criss-cross attention for semantic segmentation,” in Proceedings of the IEEE/CVF international conference on computer vision. 603–612.

[B14] Javierto D. P. P. Martin J. D. Z. Villaverde J. F. (2021). “Robusta Coffee Leaf Detection based on YOLOv3-MobileNetv2 model,” in 2021 IEEE 13th International Conference on Humanoid, Nanotechnology, Information Technology, Communication and Control, Environment, and Management (HNICEM): IEEE. 1–6.

[B15] Ji Y. Xu J. Yan B. (2024). Coffee green bean defect detection method based on an improved YOLOv8 model. J. Food Process. Preserv. 2024, 2864052. doi: 10.1155/2024/2864052

[B16] Liu G. Zhang Y. Liu J. Liu D. Chen C. Li Y. (2024). An improved YOLOv7 model based on Swin Transformer and Trident Pyramid Networks for accurate tomato detection. Front. Plant Sci. 15, 1452821. doi: 10.3389/fpls.2024.1452821 39391778 PMC11464322

[B17] Liu W. Lu H. Fu H. Cao Z. (2023). “Learning to upsample by learning to sample,” in Proceedings of the IEEE/CVF International Conference on Computer Vision.

[B18] Liu Y. Shao Z. Hoffmann N. (2021). Global attention mechanism: Retain information to enhance channel-spatial interactions.

[B19] Lu H. Liu W. Fu H. Cao Z. (2022). “FADE: Fusing the assets of decoder and encoder for task-agnostic upsampling,” in European Conference on Computer Vision: Springer. 231–247.

[B20] Redmon J. Divvala S. Girshick R. Farhadi A. (2016). “You only look once: Unified, real-time object detection,” in Proceedings of the IEEE conference on computer vision and pattern recognition. 779–788.

[B21] Ren S. He K. Girshick R. Sun J. (2016). Faster R-CNN: Towards real-time object detection with region proposal networks. IEEE transactions on pattern analysis and machine intelligence 39, 1137–1149. doi: 10.1109/TPAMI.2016.2577031 27295650

[B22] Shen Z. Zhang M. Zhao H. Yi S. Li H. (2021). “Efficient attention: Attention with linear complexities,” in Proceedings of the IEEE/CVF winter conference on applications of computer vision. 3531–3539.

[B23] Sirisha U. Praveen S. P. Srinivasu P. N. Barsocchi P. Bhoi A. K. (2023). Statistical analysis of design aspects of various YOLO-based deep learning models for object detection. Int. J. Comput. Intell. Syst. 16, 126. doi: 10.1007/s44196-023-00302-w

[B24] Sunkara R. Luo T. (2022). “No more strided convolutions or pooling: A new CNN building block for low-resolution images and small objects,” in Joint European conference on machine learning and knowledge discovery in databases: Springer. 443–459.

[B25] Unal Y. Taspinar Y. S. Cinar I. Kursun R. Koklu M. (2022). Application of pre-trained deep convolutional neural networks for coffee beans species detection. Food Anal. Methods 15, 3232–3243. doi: 10.1007/s12161-022-02362-8

[B26] Valles-Coral M. A. Ivan Bernales-del-Aguila C. Benavides-Cuvas E. Cabanillas-Pardo L. (2023). Effectiveness of a cherry coffee sorter prototype with image recognition using machine learning. Rev. Bras. Ciências Agrárias - Braz. J. Agric. Sci. 18. doi: 10.5039/agraria.v18i1a2586

[B27] Vegro C. L. R. de Almeida L. F. (2020). “Global coffee market: Socio-economic and cultural dynamics,” in Coffee consumption and industry strategies in Brazil. Elsevier. 3–19.

[B28] Velásquez S. Franco A. P. Peña N. Bohórquez J. C. Gutiérrez N. (2021). Classification of the maturity stage of coffee cherries using comparative feature and machine learning. Coffee Sci. 16, e161710–e161710. doi: 10.25186/.v16i.1710

[B29] Wang J. Chen K. Xu R. Liu Z. Loy C. C. Lin D. (2019). “Carafe: Content-aware reassembly of features,” in Proceedings of the IEEE/CVF international conference on computer vision. 3007–3016.

[B30] Wang X. Girshick R. Gupta A. He K. (2018). “Non-local neural networks,” in Proceedings of the IEEE conference on computer vision and pattern recognition. 7794–7803.

[B31] Wang C. Han Q. Li C. Zou T. Zou X. (2024). Fusion of fruit image processing and deep learning: a study on identification of citrus ripeness based on R-LBP algorithm and YOLO-CIT model. Front. Plant Sci. 15, 1397816. doi: 10.3389/fpls.2024.1397816 38903428 PMC11188418

[B32] Wang Q. Wu B. Zhu P. Li P. Zuo W. Hu Q. (2020). “ECA-Net: Efficient channel attention for deep convolutional neural networks,” in Proceedings of the IEEE/CVF conference on computer vision and pattern recognition. 11534–11542.

[B33] Woo S. Park J. Lee J.-Y. Kweon I. S. (2018). “Cbam: Convolutional block attention module,” in Proceedings of the European conference on computer vision (ECCV). 3–19.

[B34] Zhang Z. Lei X. Huang K. Sun Y. Zeng J. Xyu T. (2024). Multi-scenario pear tree inflorescence detection based on improved YOLOv7 object detection algorithm. Front. Plant Sci. 14, 1330141. doi: 10.3389/fpls.2023.1330141 38317836 PMC10840500

[B35] Zhu X. Chen F. Zheng Y. Chen C. Peng X. (2024). Detection of Camellia oleifera fruit maturity in orchards based on modified lightweight YOLO. Comput. Electron. Agric. 226, 109471. doi: 10.1016/j.compag.2024.109471

[B36] Zhu Z. Xu M. Bai S. Huang T. Bai X. (2019). “Asymmetric non-local neural networks for semantic segmentation,” in Proceedings of the IEEE/CVF international conference on computer vision. 593–602.

